# Diagnostic value of *PPARδ* and miRNA-17 expression levels in patients with non-small cell lung cancer

**DOI:** 10.1038/s41598-021-03312-w

**Published:** 2021-12-17

**Authors:** Monika Migdalska-Sęk, Barbara Modrzewska, Jacek Kordiak, Dorota Pastuszak-Lewandoska, Justyna M. Kiszałkiewicz, Filip Bielec, Adam Antczak, Ewa Brzeziańska-Lasota

**Affiliations:** 1 Department of Biomedicine and Genetics, Medical University of Lodz, St. Pomorska 251, C-5, 92-213 Lodz, Poland; 2 Clinic of Thoracic Surgery, General and Oncological Surgery University Clinical Hospital Named After the Military Medical Academy - Central Veterans’ Hospital, Medical University of Lodz, St. Żeromskiego 113, 90‑549 Lodz, Poland; 3 Department of Microbiology and Laboratory Medical Immunology, Medical University of Lodz, St. Pomorska 251, C-5, 92-213 Lodz, Poland; 4 Department of General and Oncological Pulmonology, Medical University of Lodz, St. Kopcińskiego 22, 90-153 Lodz, Poland

**Keywords:** Cancer, Genetics, Molecular biology, Biomarkers, Medical research

## Abstract

The *PPARδ* gene codes protein that belongs to the peroxisome proliferator-activated receptor (PPAR) family engaged in a variety of biological processes, including carcinogenesis. Specific biological and clinical roles of *PPARδ* in non-small cell lung cancer (NSCLC) is not fully explained. The association of PPARα with miRNA regulators (e.g. miRNA-17) has been documented, suggesting the existence of a functional relationship of all PPARs with epigenetic regulation. The aim of the study was to determine the *PPARδ* and miR-17 expression profiles in NSCLC and to assess their diagnostic value in lung carcinogenesis. *PPARδ* and miR-17 expressions was assessed by qPCR in NSCLC tissue samples (n = 26) and corresponding macroscopically unchanged lung tissue samples adjacent to the primary lesions served as control (n = 26). *PPARδ* and miR-17 expression were significantly lower in NSCLC than in the control (p = 0.0001 and p = 0.0178; respectively). A receiver operating characteristic (ROC) curve analysis demonstrated the diagnostic potential in discriminating NSCLC from the control with an area under the curve (AUC) of 0.914 for *PPARδ* and 0.692 for miR-17. Significant increase in *PPARδ* expression in the control for current smokers vs. former smokers (p = 0.0200) and increase in miR-17 expression in control tissue adjacent to adenocarcinoma subtype (p = 0.0422) were observed. Overexpression of miR-17 was observed at an early stage of lung carcinogenesis, which may suggest that it acts as a putative oncomiR. *PPARδ* and miR-17 may be markers differentiating tumour tissue from surgical margin and miR-17 may have diagnostic role in NSCLC histotypes differentiation.

## Introduction

Peroxisome proliferator-activated receptors (PPARs) are transcription factors that belong to the family of nuclear hormone receptors and are involved in metabolic and developmental processes^1–3^. To date, three PPAR isotypes have been identified: α, β/δ (β in amphibians, and δ in mammals) and γ, produced by different genes, displaying different expression profiles in tissues and involved in various cellular processes^1,4^. The most common isotype is PPARβ/δ expressed in all tissues, but its role in cells is poorly understood, because it is less investigated that the others^4,5^. It is known, however, that it is involved in the control of energy homeostasis, thermogenesis, cell proliferation and differentiation, lipid and glucose metabolism, as well as in the transport of cholesterol^1,2,4,6^. Because of that PPARβ/δ is considered a major regulator of metabolic disorders, such as obesity, dyslipidaemia, type 2 diabetes mellitus, and non-alcoholic fatty liver disease^6,7^. Moreover, it is involved in wound healing and regeneration^2,8^, increases insulin sensitivity^9^, as well as inhibits the inflammatory process^10–12^. PPARδ also lowers oxidative stress and prevents symmetric cell division, thus increases the endurance capacity of muscle cells and prevents exhaustion of hematopoietic stem cells^13^.

Some studies indicate that it increases the growth of cancer (liver, intestine, breast, prostate and lung); it is observed to be overexpressed in many human cancers, therefore it can be important in the initiation and progression of tumours^13^. Moreover, the increase in its expression is correlated with poor survival of patients with various types of cancers^14^. However, there are also negative claims of this *PPARδ* function^9^. Thus, the role of PPARδ in cancer is probably dependent on the cell type, differentiation stage, cellular context and the microenvironment of soluble mediators (e. g. ligands)^14^. For example, in the case of skin cancer researchers tend to agree that PPARβ/δ plays a protective role in tumour progression and metastasis^10,15,16^. *PPARβ/δ* can also regulate angiogenesis, which is an important process in the development of cancer, because new blood vessels appearing in the area of tumours create appropriate conditions for their development^2,4,17^. The inhibition of angiogenesis may reduce the growth rate of primary tumours and the possibility of metastasis^4^. PPARδ may also affect the development of cancer, because it can promote terminal differentiation in keratinocytes, intestinal epithelium, oligodendrocytes and osteoblasts^9^.

Literature data suggest the involvement of PPARδ in the proliferation of non-small cell lung carcinoma (NSCLC), which is the most common form of lung cancer and the most common cause of death among oncological patients^1,2,14^. On the other hand, a correlation between a higher *PPARδ* expression and a quick recurrence of lung adenocarcinoma (AC) was observed^14^. Some other studies also imply that PPARβ/δ plays a protective role against lung cancer^10^.

Literature data suggest that for the *PPARδ* gene, one of the putative regulatory miRNAs is miR-17-5p^3,18,19^. Based on the 3'-UTR complementary prediction with Target Scan Human 7.2. database (http://www.targetscan.org/vert_72/), it is evident that many genes are potential targets of miR-17-5p. Due to the similarity of miR-17-5p sequence to the 7mer-A1 region in 3′UTR of *PPARδ*, the above-mentioned databases suggest the participation of miR-17-5p also in the regulation of *PPARδ* expression. Previous studies on the mechanism of regulation of miR-17 by the nuclear PPAR-β/δ receptor have shown its participation in the suppression of pathogenic activation of inflammation caused by hypoxic-ischaemic state and anti-apoptotic activity in neurons^3,18^. Importantly, *PPARδ* has been identified as a direct target of miR-17 in neuroblastoma^19^, and the widely studied relationship between miR-17–92 cluster encoding miR-17-5p and carcinogenesis allows the assumption of co-expression between this miRNA and *PPARδ* in NSCLC. In order to be able to assess whether changes in *PPARδ* gene expression, important for tumour pathogenesis, including NSCLC, are related to changes in miR17-5p level, it is necessary to analyze the correlation between the expression level of both molecules. So far, the ENCORI (The Encyclopedia of RNA Interactomes) website (https://starbase.sysu.edu.cn/) presents the interactions of miRNA-target gene (miR-17-5p – *PPARδ*) in NSCLC, and based on it the co-expression profile of *PPARδ* and miR-17-5p in 512 lung adenocarcinoma (LUAD) samples with p = 0.981 and in 475 lung squamous cell carcinoma (LUSC) samples with p = 0.00847 was assessed.

MicroRNAs (miRNAs) are short non-coding RNAs that are involved in regulation of gene expression, which may act as tumour oncogenes or suppressors, because they may regulate cell growth, proliferation, differentiation and apoptosis^3,20^. They may potentially serve as a diagnostic and / or prognostic marker in the course of different cancers^20^. One of such microRNA is miR-17, which takes part in cell proliferation and apoptosis, the development of some human organs (heart, lung) and the immune system^3,20^. It has been shown that it negatively regulates the stability of thioredoxin-interacting protein (TXNIP) mRNA and inhibits ROS generation in human microglial cells^3^. It is observed to be aberrantly expressed in various types of cancer and the increase in its expression is correlated with poor survival of patients^20,21^. However, the results of studies on its role in lung carcinogenesis are divergent. According to some authors, it is one of microRNAs that promotes cell proliferation in lung cancer^21,22^, but others claim that it inhibits the growth, migration and invasion of NSCLC cells^23^. Heegaard et al.^24^ observed a significant reduction of miR-17 in the serum of patients with NSCLC, suggesting its tumour-suppressive role in this type of cancer. Similarly, Li et al.^25^ demonstrated its downregulation in NSCLC tissues, which was even greater in more advanced pathological stages of cancer (T2-T4 comparing with T1). Furthermore, those authors noted that miRNA-17 inhibits proliferation and triggers apoptosis in NSCLC cells. Also in other type of cancer, prostate cancer, Ottman et al.^26^ observed that miR-17 decreases proliferation of cells, tumour growth and delays tumourigenicity in animals.

In the light of the controversial results, further study needs to be conducted to clarify an influence of *PPARδ* gene and miR-17 on carcinogenesis process in lung cancer. Thus, present study has evaluated changes in the expression of the *PPARδ* and miR-17 in the tissue samples collected from patients with NSCLC. The purpose of the study was also to analyse the correlation of the expression levels of the *PPARδ* and the miRNA-17 with the clinical features of patients and to determine whether the relationship between *PPARδ* gene and miR-17 expression exists.

## Materials and methods

### Patients and tissue collection

Twenty six (26) adult patients with NSCLC were qualified for the study—11 women and 15 men, aged 51 to 81 (mean age 66.96 ± 7.95 years). In patients, lung resection (pulmonectomy or lobectomy) was performed at the Department of Thoracic Surgery, General and Oncologic Surgery, Military Medical Academy Memorial Teaching Hospital of The Medical University of Lodz – Central Veterans’ Hospital, Lodz, Poland, between 2018 and 2019. This study was conducted in accordance with Good Clinical Practice and the principles of the Helsinki Declaration. The protocols of this study were approved by the Bioethics Committee of the Medical University in Lodz (resolution No. KE/745/18, 12 June 2018). All participants signed an individual consent form for participation in the study.

For analysis, lung tissue samples (approximately 100 mg) were collected from primary lesion and surgery margin (2 cm away from the primary lesion), as a control group (macroscopically unchanged lung tissue). The resected primary tumours were post-operatively subjected to the histopathological analysis. NSCLC samples were classified as: squamous cell carcinoma (SCC) and adenocarcinoma (AC). All cases were primary tumours without chemo-or radiotherapeutic treatment. The stage of cancer was established according to the TNM classification and the AJCC classification system^27^. Patients were divided into groups depending on the members of pack-years (PY, 1 Pack Year = 20 cigarettes smoked per day for 1 year; according to NCI Dictionary of Cancer Terms)^28^. Patient demographic characteristics and features of lung cancer are shown in Table 1.

**Table 1:** Demographic characteristics of patients and features of lung cancer.

Patients’ characteristics	Number of patients (%)
*Gender*	
Women	11 (42)
Men	15 (58)
*Age (years)*	
≤ 65	12 (46)
> 65	14 (54)
*Smoking history*	
Former smokers	11 (42)
Current smokers	14 (54)
Non-smokers	1 (4)
*Pack-years*	
≤ 40 PYs	13 (52)
> 40 PYs	12 (48)

Tumours’ characteristics	Number of cases (%)
*Histopathological subtype*	
SCC	14 (54)
AC	12 (46)
*Tumour size (TNM classification)*	
T1	7 (27)
T2	15 (58)
T3	4 (15)
*Presence of metastasis (TNM classification)*	
N0	15 (58)
N1	9 (35)
N2	2 (7)
*AJCC staging*	
I	11 (42)
II	11 (42)
III	4 (15)

### RNA isolation, qualitative and quantitative RNA evaluation

Lung tissue samples were placed in fixRNA buffer (Eurx, Gdańsk, Poland), then divided into smaller parts, homogenized and frozen at -80 °C until use. Isolation of total RNA from tissue homogenates was performed using the mirVana™ miRNA Isolation Kit (Life Technologies, Carlsbad, CA, USA) according to the manufacturer's protocol. Qualitative and quantitative evaluation of the isolated RNA was performed by spectrophotometric method by measuring the absorbance with the Eppendorf BioPhotometerTM Plus apparatus (Eppendorf, Hamburg, Germany), at 260/280 nm wavelengths. Prepared RNA was divided into portions and frozen at -80 °C until the real-time polymerase chain reaction (qPCR) was performed.

### Evaluation of gene/miRNA expression

The reverse transcription (RT) reaction for genes was performed using the High-Capacity cDNA Reverse Transcription Kit (Applied Biosystems, USA), in a volume of 20 μl. The reaction mixture contained: 10 × RT buffer, 25 × dNTP Mix (100 mM), 10 × RT Random Primers, MultiScribe™ Reverse Transcriptase (50 U/µL), RNase Inhibitor and nuclease-free water. 100 ng of total RNA was added to the reaction mixture. The negative control in the RT reaction was carried out using water instead of RNA. The following RT reaction conditions were used: 10 min at 25 °C, 120 min at 37 °C, 5 min at 85 °C and cooling at 4 °C.

The reverse transcription (RT) for miRNA of 5 μl (10 ng) of total RNA in a 15-μl reaction was carried out using a TaqMan® MicroRNA Reverse Transcription Kit (Applied Biosystems, Carlsbad, CA). RT master mix contained: 25 × dNTP Mix (100 mM), MultiScribe™ Reverse Transcriptase (50 U/µL), 10 × RT buffer, RNase Inhibitor (20 U/µL), nuclease-free water and the specific RT primers (small RNA-specific RT primers) included in individual TaqMan® MicroRNA Assays: hsa-miR-17-5p (CAAAGUGCUUACAGUGCAGGUAG) and RNU6B (CGCAAGGATGACACGCAAATTCGTGAAGCGTTCCATATTTTT) as endogenous control (Applied Biosystems, Carlsbad, CA). RT reaction was performed in a Personal Thermocycler (Eppendorf, Germany) in the following conditions: 30 min at 16 °C, followed by 30 min at 42 °C, then the samples were heated to 85 °C for 5 min, and held at 4 °C. RT products were stored at -20° C until further analysis.

Relative gene/miRNA expression was assessed by real-time polymerase chain reaction (qPCR) using a 7900HT Fast Real-Time PCR System apparatus (Applied Biosystems, Carlsbad, CA). A total reaction mixture volume of 20 μl contained: cDNA (1–100 ng), KAPA PROBE FAST qPCR Master Mix (2X) ABI Prism™ (Kapa Biosystems Ltd, London, UK), RNase-free water and 20xTaqMan® Gene Expression Assay for the following genes: *PPARδ* (Hs00987008_m1), and *ACTB* (Hs99999903_m1) selected as the reference gene in the qPCR reaction. Assays for the following miRNA: *miRNA-17*, and *RNU6B* (endogenous control) were used in the qPCR reaction. The relative expression levels of the analyzed gene/miRNA were evaluated by the delta-delta CT method (TaqMan Relative Quantification Assay software, Applied Biosystems) and presented as RQ values relative to the *ACTB/RNU6B* reference gene/miRNA, respectively. The following formula was used to determine the ΔΔCT value: ΔΔCT = ΔCT test sample—ΔCT calibrator sample. For calibrator (commercial sample—Human Lung Total RNA, Invitrogen™), RQ value was considered equal to 1. The obtained results were compared in terms of the NSCLC histopathological subtype, cancer stage (TNM, AJCC), age of patients, gender and smoking history. In the case of the test samples, the increased expression was recognized when the RQ value was more than 1 and the decreased expression—when the RQ value was less than 1.

### Statistical analysis

Statistical analysis was performed using Statistica for Windows 10.0 software (StatSoft, Cracow, Poland) (v.13). In order to check the occurrence of statistical significance between the analyzed groups, Mann–Whitney U-test and was used. The Spearman rank correlation coefficient was used to measure the direction and strength of the relationship for individual variables. To assess the specificity and sensitivity of *PPARδ* and miR-17 as potential diagnostic predictors that could differentiate NSCLC from the operating margin, the receiver operating characteristic (ROC) curve was analyzed and the area under the curve (AUC) was resolved with a 95% confidence interval (CI). The results of relative expression analysis (RQ value) are presented as median values. Statistical significance was determined at p < 0.05.

### Ethical approval

The manuscript has not been previously published or submitted in whole or in part elsewhere. This study was conducted in accordance with Good Clinical Practice and with the 1964 Declaration of Helsinki and its later amendments or comparable ethical standards. All analysis performed in studies involving human tissue samples were approved by the Bioethics Committee of the Medical University in Lodz (resolution No. KE/745/18, 12 June 2018).

### Informed consent

Informed consent was obtained from all patients included in the study.

## Results

### Relative gene/miRNA expression in NSCLC tissue vs. controls

**Figure 1:**
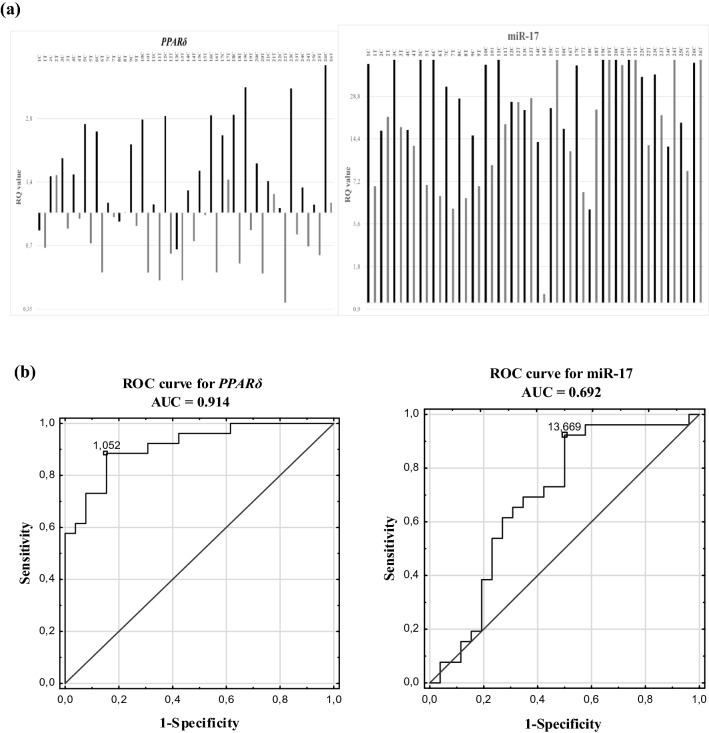
*PPARδ* and miR-17 in NSCLC tissue and macroscopically unchanged tissue groups: (**a**) the results of qPCRs presented as log2 (RQ) of all studied samples (C—control tissue, T—tumour tissue); (**b**) receiver operating characteristic (ROC) curve analysis.

The relative expression level of *PPARδ* and miR-17 in NSCLC tissues *vs.* control tissues was compared. The obtained results showed a decreased expression of *PPARδ* in 81% of NSCLC samples and its increased expression in 88% of control samples. The RQ value of miR-17 was increased in all samples, both NSCLCs and controls. Statistically significant difference in the relative expression of *PPARδ* and miR-17 between the NSCLC samples and the control tissue (p = 0.0001 and p = 0.0178; respectively, Mann–Whitney *U* test) was observed. The obtained results are presented in Fig. 1a and Table 2.

**Table 2:** Expression levels of *PPARδ* and miR-17, presented as median RQ values, and the number of samples with the decreased/increased expression in the studied groups.

Tissue	*PPARδ*			miRNA-17		
	RQ value	Number (%) of samples		RQ value	Number (%) of samples	
		RQ < 1	RQ > 1		RQ < 1	RQ > 1
NSCLC	0.724	21 (81)	5 (19)	15.226	0 (0)	26 (100)
Control	1.648	3 (12)	23 (88)	36.608	0 (0)	26 (100)

We also evaluated the potential of the *PPARδ* and miR-17 as diagnostic classifiers for NSCLC by performing receiver operating characteristic curves and area under the curve (ROC-AUC) analyses. The obtained results revealed that *PPARδ* and miR-17 expressions were able to differentiate macroscopically unchanged lung tissue from NSCLC with AUC-ROC values of 0.914 (95% CI: 0.840–0.989; p = 0.00001) and 0.692 (95% CI: 0.840–0.989, p = 0.0117), respectively. Both the specificity and sensitivity of *PPARδ* in tissue type prediction were 84.6%, while the specificity of miR-17 was 88.5% and its sensitivity was 50% (see Fig. 1b).

### Gene/miRNA RQ values vs. clinicopathological parameters

The obtained RQ values for *PPARδ* gene and miR-17 were analyzed in relation to clinical features of patients: age at time of diagnosis, gender and smoking history as well as histopathological characteristics of tumours (according to pTNM and AJCC classifications and NSCLC subtypes). Table 3a, b indicates RQ values (medians) of the studied gene/miRNA in relation to the mentioned clinicopathological parameters.

**Table 3:** *PPARδ* (a) and miR-17 (b) expression levels (median RQ values) in individual NSCLC tissue group and control group.

Features	NSCLC tissue	p-value Mann Whitney U-test	Macroscopically unchanged tissue (control)	p-value Mann Whitney U-test
*(a) PPARδ*				
≤ 65 years	0.816	0.5892	1.959	0.8170
> 65 years	0.668		1.451	
Women	0.842	0.2535	1.414	0.1611
Men	0.643		2.430	
Former smokers	0.791	0.1627	1.368	0.0200*
Current smokers	0.637		2.528	
≤ 40 PYs	0.683	0.2888	1.517	0.1086
> 40 PYs	0.753		2.528	
SCC	0.688	0.3961	1.502	0.9385
AC	0.842		1.762	
T1	0.828	0.7771	1.811	0.6029
T2 + T3	0.692		1.517	
N0	0.732	0.9594	1.714	0.6404
N1 + N2	0.692		1.414	
I	0.732	0.7988	1.714	0.6404
II + III	0.692		1.517	
*(b) miR-17*				
≤ 65 years	12.949	0.5892	44.369	0.4875
> 65 years	19.484		26.996	
Women	12.881	0.6038	33.655	0.9586
Men	20.704		39.561	
Former smokers	20.704	0.8481	27.760	0.5290
Current smokers	15.226		43.933	
≤ 40 PYs	11.836	0.1211	23.748	0.6833
> 40 PYs	22.200		40.288	
SCC	20.968	0.2076	24.638	0.0422*
AC	9.319		50.615	
T1	13.017	0.9550	39.561	0.9539
T2 + T3	18.264		33.655	
N0	12.881	0.3302	33.655	0.7555
N1 + N2	21.232		41.016	
I	13.017	0.8785	39.561	0.5681
II + III	18.264		27.760	

Statistically significant p < 0.05.

There was no significant correlation between RQ values of gene/miRNA and patients’ age (two age groups: ≤ 65 years and > 65 years) and gender (p > 0.05; Mann–Whitney *U* test). Differences in the level of expression of the *PPARδ* gene depending on the history of cigarette smoking (tobacco addiction and consumption) were observed, but only in the control group. A significant increase in the RQ value of *PPARδ* in the control tissue was demonstrated in the case of patients who were still smoking (current smokers) *vs.* those who stopped smoking (former smokers) (p = 0.0200; Mann–Whitney *U* test). We did not observe such a relationship for miR-17 (p > 0.05; Mann–Whitney *U* test). In NSCLC group, the level of gene/miRNA expression was lower among current smokers compared to the patients who stopped smoking, but without statistical significance (p > 0.05; Mann–Whitney *U* test).

No significant differences in RQ values *PPARδ* gene and miR-17 depending on the number of cigarettes smoked out, presented in pack years (≤ 40 vs. > 40PY) was showed (p > 0.05; Mann–Whitney *U* test). In addition, an analysis was carried out in the entire group of smokers to see if there is a correlation between *PPARδ*/miRNA-17 expression and the amount of cigarettes smoked in relation to the length of the smoking (PYs). Spearman’s rank correlation revealed rho = 0.0996 for *PPARδ* (p = 0.6359) and rho = 0.3867, on the border of statistical significance for miR-17 (p = 0.0562).

**Figure 2:**
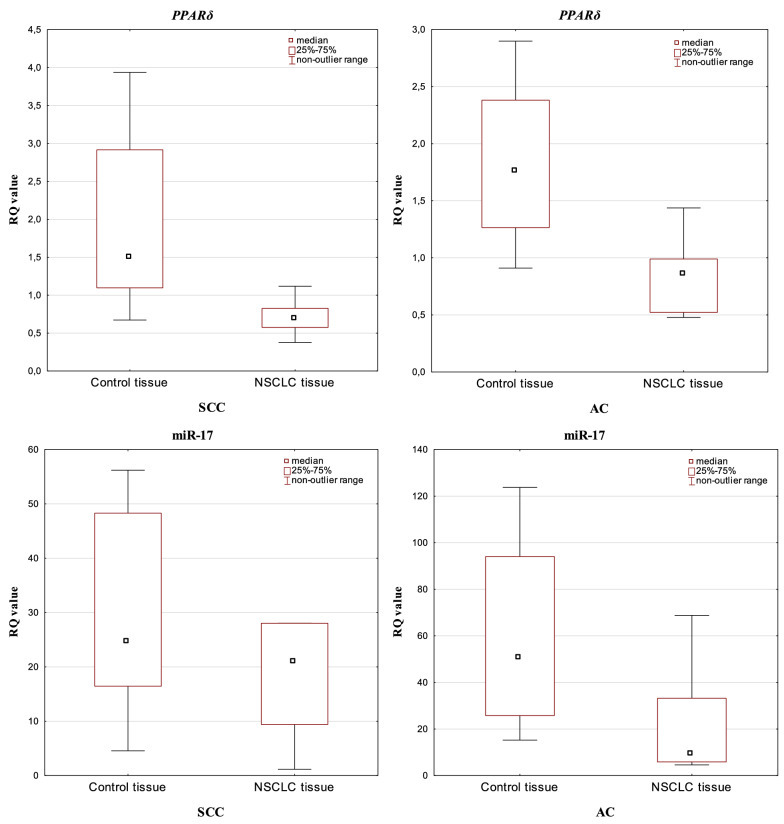
Box-and-whisker plots representing *PPARδ* and miRNA-17 expression levels (median RQ values) in NSCLC and control tissue depending on the histopathological subtypes (SCC and AC).

Depending on the NSCLC histopathological diagnosis, dividing the study group on SCC and AC, significant differences in the level of *PPARδ* and miR-17 expression were not observed in tumour samples (p > 0.05; Mann–Whitney *U* test). Analysis performed between histological subtypes in macroscopically unchanged lung tissue samples revealed statistically significant increase in miRNA-17 expression in AC group (p = 0.0422; Mann–Whitney *U* test). Comparison of the *PPARδ* and miR-17 expression levels between NSCLC and control tissue in the studied histotypes showed significantly lower expression values of both *PPARδ* and miR-17 in the tumour tissue in the AC group (p = 0.0004 and p = 0.0120; respectively, Mann–Whitney *U* test). In SCC, a significant difference in the level of expression between tumour and control tissue was observed for *PPARδ* (p = 0.0003; Mann–Whitney *U* test) (see Fig. 2).

There were no statistically significant differences in the level of *PPARδ* and miR-17 depending on tumour staging according to pTNM and AJCC classifications (p > 0.05; Mann–Whitney *U* test).

### Correlation between the expression level of PPARδ gene and miRNA-17

**Figure 3:**
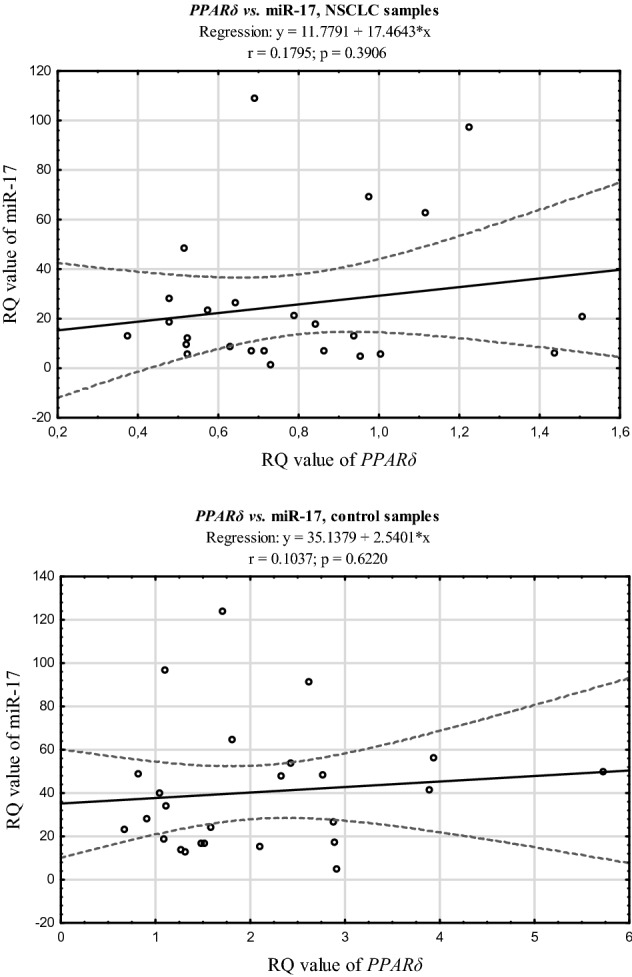
The scatter plots showing correlations between expression levels (RQ values) of *PPARδ* and miRNA-17 in NSCLC and control tissue.

The simultaneous decreased expression level of *PPARδ* and increased expression level of miRNA-17 were observed in 21 NSCLC tissue samples (81%) and in 3 control tissue samples (12%). There were no statistically significant correlations between the expression of studied *PPARδ* and miR-17 neither in the NSCLC nor in tumour tissue margins (p > 0.05; Spearman’s rank correlation coefficient) (see Fig. 3).

## Discussion

Peroxisome proliferator-activated receptors, PPARs, are transcription factors whose main role is to control fatty acid metabolism and maintain glucose homeostasis, as well as to participate in cell proliferation and differentiation. To date, three PPAR isotypes have been identified: α, β/δ and γ. Each of them is a product of a separate gene, has a different expression profile in tissues, is activated by specific ligands and is involved in various, although often complementary, cellular processes. The most common isotype is PPARβ/δ expressed in all tissues at a similar level. So far, it has been demonstrated that it is involved in the control of energy homeostasis and thermogenesis. It is also involved in the regulation of fatty acid β-oxidation and cholesterol transport, which is why its ligands are proposed as drugs in the treatment of metabolic syndrome X. In addition, it plays an important role in the proliferation of keratinocytes and the process of wound healing^4^. In contrast to these roles of PPARβ/δ established in normal physiology, the effect of PPARβ/δ in carcinogenesis remains still controversial. Previous studies have confirmed the relationship of *PPARβ/δ* with lung cancer^2,10,29^, but the mechanism of its effects has not been determined.

The aim of our study was to determine the level of the *PPARδ* gene expression in patients with NSCLC. The control material was a fragment of the macroscopically unchanged lung tissue surrounding the tumour from the same patient. To the best of our knowledge, we were the first to observe significant differences between the expression of *PPARδ* in the lung tumour and control tissue. In addition, we confirmed the high sensitivity and specificity of the *PPARδ* potential for prediction of type of tissue. Such a difference indicate the possible utility of *PPARδ* gene expression level as a diagnostic marker. Literature data describing expression of *PPARβ/δ* mRNA and/or PPARβ/δ protein in both types of tissues are inconclusive and largely dependent on the method used to determine its expression patterns^9^. Immunohistochemistry showed higher PPARβ/δ expression in tumours than in nontransformed control lung tissue, but Western blot analysis did not support this concept^9,30^. On the other hand, tissue microarray indicated moderate expression of *PPARβ/δ* in respiratory epithelial cells of the bronchus and lack or weaked expression in human lung cells carcinomas^9^. Those results are consistent with ours and the qPCR technique that we used is considered the "gold standard" in microarray validation^31^, thus supporting the diagnostic significance of *PPARδ*.

The observed decreased *PPARδ* expression in NSCLC and its increased level in adjacent normal lung tissue may indicate its putative role as tumour suppressor gene. Literature data indicate that PPAR receptors, may be the target of anti-cancer therapy in intestine, mammary gland, prostate and lymphatic system cancers, because they exert antiproliferative effects^4^. The suppressive role of *PPARβ/δ* in the lung cancer was also signaled by Peters et al.^9^ who claimed that in vast majority of human lung cancers (both SCC and AC) expression of *PPARβ/δ* was low or none. Other research, conducted on *PPARβ/δ* ligand, L165041 has proved that it inhibits human lung adenocarcinoma cell proliferation^29^, while the study performed on transgenic mouse model has showed that lack of PPARβ/δ expression is associated with exacerbation of lung cancer^32^.

On the other hand, the prooncogenic properties of *PPARδ* have also been observed. *PPARδ* gene upregulation in 3^rd^ stage of cancer and its impact on the metastasis development in various cancer models (lung, breast, colorectal) in vivo was reported^17^. Genini et al.^2^ have observed increased *PPARβ/δ* mRNA level in NSCLC comparing to the normal lung tissue and concomitantly up-regulated VEGF and components of the Cox-2/prostaglandin synthetic pathway in a subset of NSCLC, thus suggesting that activation of these pathways plays a role in lung carcinogenesis. Also on protein level the increased PPARδ expression was demonstrated in the subtypes of lung cancer, i.e., adenocarcinoma and squamous carcinoma^30^. Regarding receptors ligands, Han et al.^33^ have showed that PPARβ/δ agonist (GW501516) stimulates human lung carcinoma cell proliferation. Summarizing the above, *PPARδ* is able to modulate both cancer cells and unaltered cells in the surroundings of tumour depending on its influence on target genes^10^. Ligand-bound PPARβ/δ induces expression of target genes, while if it is not bound by its ligands it can repress the transcription of its target genes. PPARβ/δ activity may also be affected by the presence or absence of cofactors and repressors^10,14^. It is suggested that PPARδ plays different roles depending on the site of its expression: in normal cells in the tumour microenvironment it causes promotion of tumourigenesis, while in cancer cells – its suppression^34,35^. It seems to be consistent with the findings of our research.

Considering the relationship between the status of *PPARδ* expression and the clinical and pathological features of NSCLS, in our research we didn’t observe any differences in *PPARδ* expression depending on the gender or age of the subjects, the number of cigarettes smoked out presented in pack years, the stage of the cancer according to TNM and AJCC classifications, as well as the histological subtype of the cancer (SCC *vs.* AC). To our knowledge, there are no published studies comparing the expression of *PPARδ* with the above mentioned features, apart from Pedchenko et al.^30^ who, with the use of the immunochemical method, also didn’t notice any significant correlation between the level of *PPARδ* and history of smoking, tumour stage or tumour histology.

However, we observed significantly higher *PPARδ* expression in unchanged lung tissue of current smokers comparing to former smokers. This result seems to confirm the *PPARβ/δ* activation mechanism proposed by Sun et al.^1^ who showed a time- and dose-dependent induction of PPARβ/δ protein by nicotine through nicotinic acetylcholine receptor nAChR–mediated activation of PI3K/mTOR pathway. The role of *PPARβ/δ* in mediating the effect of nicotine on the growth of cancer cells is simultaneously suggested^1^. In our study, such a role of *PPARδ* is visible already at an early stage of carcinogenesis and may be the result of the genotoxic action of oxidative stress which leads to early molecular changes. We did not find any other research comparing expression of *PPARδ* depending on smoking status.

Regarding miR-17, the literature data confirm that miR-17–92 cluster encoding miR-17-5p and miR-17-3p is necessary for normal lung development and alterations in its expression have been reported in various pulmonary diseases, such as lung cancer^25,36^. High expression of miR-17 in lung cancerous tissue was observed by Saito et al.^37^. Similarly, Chen et al.^21^ noted elevated expression levels in tumour tissue and also in serum of patients with lung cancer. Our results confirmed overexpression of miR-17 in all NSCLC and control tissues samples, however, with significantly lower median RQ value in NSCLC. A 15-fold increase in miR-17 expression in NSCLC and a 30-fold increase in miR-17 expression in tissue surrounding the tumour seems interesting and speaks for an oncogenic role of miR-17. Current publications, however, are not conclusive as to the functional mechanism of the miR-17 and the results are inconsistent. Cloonan et al.^38^ have shown that miR-17-5p may be either an oncogene or a tumour suppressor gene in different cell environments, depending on the expression of other transcriptional regulators. MiR-17-5p acts specifically at the G1/S-phase cell cycle boundary, by targeting more than 20 genes, both pro- and anti-proliferative, involved in the transition between these phases^38–40^.

In our study, we observed differences between the expression of miR-17 and the stage of cancer according to the TNM and AJCC classification, showing its higher expression in more advanced stages of cancer, however those results were statistically insignificant. We noted that T2 and T3 tissue samples were characterized by a comparable expression of miR17, higher than T1. Increased expression of miR-17 was observed in patients with stage II and III cancer, and this elevated level increased with the presence of histologically confirmed lymph node metastasis: higher expression was noted in patients with N1 + N2 feature. This result is in accordance with oncogenic properties of the miR-17, belonging to miR-17–92 cluster, which overexpression promotes cell proliferation and progression of various cancers including NSCLC^41–44^. The degree of miR-17-5p overexpression correlated with lung cancer aggressiveness, metastasis status in the lung cancer patients and responsiveness to chemotherapeutics^42,45^. A possible mechanism by which miR-17 is involved in carcinogenesis as the classical oncogene is an enhancement of cell proliferation through modulation of the PI3K/Akt/mTOR pathway^46^.

However, there are also reports supporting the suppressor role of miR-17. For example, downregulation of miRNA-17-5p in NSCLC tissues and cell lines comparing to the healthy controls was noted^25^; lower miR-17-5p expression was also observed in lung adenocarcinoma initiating cells^47^. However, miR-17-5p may play different roles at different stages of lung cancer^25^ which might explain, to some extent, the discrepant results. Additionally, the profile of miR-17 expression appears to be determined by the biological material which is analyzed. For instance, in serum mir-17 expression pattern does not reflect the pattern observed in lung tissue. Similarly to our results, the lack of statistical significance of miR-17 expression between I and II-IV stage of NSCLC according to AJCC classification was observed by Qi et al.^48^, but increased expression referred to the early stages of tumour development. Moreover, significantly higher expression of miRNA-17 in the serum of patients with stage I NSCLC comparing to healthy individuals suggests that this microRNA may be a biomarker for diagnosis of early-stage NSCLC^48^. In the light of our results and literature data, a suggestion that the tumourigenic or tumour-suppressive functions of miR-17-5p might depend on the cellular context, that is, on the model system used, cell type or cancer stage seems to be right^39,42^.

A possibility of using of miR-17 as potential diagnostic tool in lung carcinogenesis has been discussed. The results of our study have shown that the level of miR-17 expression varies depending on the NSCLC histopathological subtype. It could speak in favour for a potential diagnostic function of miR-17 in NSCLC subtype differentiation. The results of others also support such a role^44,49–51^. Interesting is that we obtained opposite results in cancerous and non-cancerous tissue for the both subtypes we examined. Such a change in the expression pattern of miRNA-17 may result from the high heterogeneity of tumour cells within the histological subtypes^52^. It may also be related to the fact that histopathological assessment does not always allow the detection of small clusters and individual tumour cells despite the finding of a negative histological margin, and transcript level dysregulation may be a sign of the ongoing process of neoplastic transformation in the margins surrounding the tumour^53^.

Comparative analyses between AC and SCC revealed upregulation of miR-17 in AC not only in solid tumour^44,50^, but also in plasma of NSCLC patients^45^. Although in our study a significant increase in the expression of miR-17 in the group of patients with AC was only observed in the tissue surrounding the tumour, the twofold change in the level of expression of miR-17 between AC and SCC, both in non-cancerous samples and tumour cells should be emphasized. This result provides evidence that miR-17 expression analysis could be useful as a support tool in NSCLC histopathological differential diagnosis. It remains to assume that, in accordance with previous reports^50,51^, SCC and AC employ different molecular pathways during their development and/or progression. There is therefore a need for separate studies evaluating the biological effect of miR-17 on these two major NSCLC subtypes.

Based on the assumption that *PPARδ* may be a molecular target for miR-17, analogously to *PPAR*α^38,54^ we have evaluated the correlation between the mRNA level of miR-17 and *PPARδ*. Numerous reports indicate that miRNAs directly target PPARs’ mRNA or indirectly affect their expression^55–57^. To the best of our knowledge, so far only two articles have shown an increased expression of nuclear *PPARβ/δ* with simultaneous activation of miR-17-5p, which correlated with a decrease in inflammation and apoptosis of neurons and the molecular mechanism has been linked to the PPAR-β/δ/miR-17/TXNIP/NLRP3 signaling pathway^3,18^. However, the mechanism of this regulation (modulating *PPARδ* expression / activity by miRNA-17) in the context of oncogenesis has not been studied. Perhaps miR-17 indirectly affects PPAR-δ expression / activity by targeting PPARs-associated cofactors and repressors, thus providing a further level of complexity in these regulatory mechanisms that we have not studied.

## Conclusions

In summary, due to the fact that we did not confirm the mutual correlation between miRNA-17 and *PPARδ* neither in the tumour tissue margins nor in NSCLC, we consider miR-17 and *PPARδ* as two independent molecular factors. The observed change in expression of miR-17 already at the margin of the tumour suggests that this miRNA may regulate basic biological processes (probably by enhancing cell proliferation) and therefore possibly plays an oncogenic role in the development of lung cancer. Our results indicate the potential role of the studied miRNA in the differentiation of NSCLC histopathological subtypes. Involvement of *PPARδ* in lung cancer biology is also undeniable, and the significant differences in expression levels between NSCLC and macroscopically unchanged lung tissue highlight its possible diagnostic role in lung cancer recognition. However, further research is required to verify the results.

### Strengths and weaknesses of the study

The strengths of this study are:

- the prospective design;
- inclusion of NSCLC patients who have not been treated with potentially mutagenic chemotherapy or radiation therapy prior to surgery;
- analysis in the most common NSCLC subtypes: AC and SCC;
- the novelty aspect in the assessment of miR-17 and *PPARδ* co-expression in NSCLC;
- analysis of *PPARδ* and miRNA-17 expression in both cancer lesion and macroscopically unchanged lung tissue (from the surgical margin).

The weaknesses of this study are:

- small size of the research group;
- lack of observation of patients with NSCLC to assess the concordance of miR-17 levels in blood and tissue;
- lack of a validation set to confirm the obtained results in the ROC curve analysis.
